# Evaluation of the Compatibility of Organosolv Lignin-Graphene Nanoplatelets with Photo-Curable Polyurethane in Stereolithography 3D Printing

**DOI:** 10.3390/polym11101544

**Published:** 2019-09-23

**Authors:** Fathirrahman Ibrahim, Denesh Mohan, Mohd Shaiful Sajab, Saiful Bahari Bakarudin, Hatika Kaco

**Affiliations:** 1 Research Center for Sustainable Process Technology (CESPRO), Faculty of Engineering and Built Environment, Universiti Kebangsaan Malaysia, Bangi 43600, Selangor, Malaysia; fatir96@gmail.com (F.I.); denesh.mohan@gmail.com (D.M.); 2 Chemical Engineering Programme, Faculty of Engineering and Built Environment, Universiti Kebangsaan Malaysia, Bangi 43600, Selangor, Malaysia; 3 Institute of Microengineering and Nanoelectronics (IMEN), Universiti Kebangsaan Malaysia, Bangi 43600, Selangor, Malaysia; saifulbahari@ukm.edu.my; 4 Kolej GENIUS Insan, Universiti Sains Islam Malaysia, Bandar Baru Nilai, Nilai 71800, Negeri Sembilan, Malaysia; hatikakaco@usim.edu.my

**Keywords:** 3D printing, composites, DLP, lignocellulose, nanoindentation

## Abstract

In this study, lignin has been extracted from oil palm empty fruit bunch (EFB) fibers via an organosolv process. The organosolv lignin obtained was defined by the presence of hydroxyl-containing molecules, such as guaiacyl and syringyl, and by the presence of phenolic molecules in lignin. Subsequently, the extracted organosolv lignin and graphene nanoplatelets (GNP) were utilized as filler and reinforcement in photo-curable polyurethane (PU), which is used in stereolithography 3D printing. The compatibility as well as the characteristic and structural changes of the composite were identified through the mechanical properties of the 3D-printed composites. Furthermore, the tensile strength of the composited lignin and graphene shows significant improvement as high as 27%. The hardness of the photo-curable PU composites measured by nanoindentation exhibited an enormous improvement for 0.6% of lignin-graphene at 92.49 MPa with 238% increment when compared with unmodified PU.

## 1. Introduction

Apart from being fast and precise, 3D printing enables a product to be easily modified and customized. This technology is suitable for applications in a field with profound individual differences, such as biomedicine [1,2]. The rise of 3D printing is also expected to increase with the use of the polymer. Resins used in stereolithography are thermosetting plastics, engineered to cure when receiving energy typically from a laser beam or ultraviolet rays [3]. The thermoset nature of stereolithography fabricated parts, with a high crosslink density, results in brittle fracture with low elongation properties [4].

Polyurethane (PU) is usually used in 3D printing because its mechanical properties can be tuned by changing its chemical structure [2]. Microbes are unable to decompose UV-curable adhesives because of their unique chemical composition and characteristics obtained after UV curing, which often lead to long-term retention issues [5]. One solution is to develop a polymer with good biodegradability via a composite approach. Composites can produce new materials with improved performance and biodegradability when a natural material is used as a filler [6].

In 2017, Malaysia accounted for 29% of the global palm oil production and 55.5% of the national export sector [7]. Despite the success of the oil palm industry, major drawbacks include the generation and utilization of empty fruit bunches (EFB). In fact, EFB has become a major threat to the industry and needs to be rectified quickly. Nevertheless, this biomass waste can be a renewable alternative to petroleum-based sources, fulfilling the energy demand and reducing pollution. Lignocellulosic materials predominantly contain a mixture of carbohydrate polymers such as cellulose, hemicelluloses, and lignin [8]. Lignin, which provides mechanical support to the cell walls of plants, also can be used to support in polymer composites. Although lignin is the second most abundant natural polymer on earth, it is under-utilized.

Lignin is categorized according to type of lignocellulose and extraction method; it can be of sulfonate or non-sulfonate type. Sulfonate lignin is produced via commercial methods such as the sulfur process and kraft process. Non-sulfur lignin is produced via the organosolv, soda, and hydrolysis processes. Extraction of lignin using sulfuric acid (H_2_SO_4_) via the kraft process is widely employed by the pulp and paper industry [9]. Organosolv is commonly utilized in laboratories owing to the easy recovery of the used acid [10]. Organosolv lignin has been utilized because of its significantly better solubility relative to those of other lignins, such as kraft and sulfur lignins [11]. Additionally, lignin produced by organosolv processes has also been reported to be high-quality, technical lignin [12]. Owing to its highly reactive polyphenolic structures, lignin can reduce the cost of producing other, more expensive, filler materials [13]; however, the sole incorporation of lignin will not boost the mechanical property of a polymer nanocomposite. The integration of filler-reinforcement of graphene also has been introduced in polymeric composites; graphene exhibited electrical, thermal, and mechanical properties as nanofiller to the reinforcement of the polymeric materials [14,15,16].

In this study, the isolation of lignin from oil palm EFB fibers was carried out using formic acid (FA) at different concentrations. The best condition in lignin isolation was further characterized by FTIR, FT-NMR, and GC-MS analyses. Subsequently, the isolated organosolv lignin was used and modified with the graphene nanoplatelets as filler-reinforcement for photo-curable PU in stereolithography 3D printing. In addition to the stress-strain behavior of the polymers, the hardness of the 3D-printed specimen was measured using nanoindentation technique for in-depth analysis of the effect of lignin-graphene compatibility in photo-curable PU resin.

## 2. Materials and Methods

### 2.1. Materials

Oil palm EFB fibers were procured from Szetech Engineering Sdn. Bhd. (Selangor, Malaysia). These were milled and sieved to obtain fibers of diameters 106–500 µm. Fractionation of lignin was performed using 90% formic acid (Merck, Darmstadt, Germany) and the lignin content was determined using 98% sulfuric acid (Merck). Graphene nanoplatelets (Sigma Aldrich, Darmstadt, Germany) was used as reinforced for the printed materials. In stereolithography 3D printing, photo-curable resin with the major composition of 45–47 wt% polyurethane acrylate, 34–36 wt% morpholine, and 15–17 wt% tripropylene glycol diacrylate was provided by Wanhao Precision Casting Co. Ltd. (Jinhua, China) and isopropyl alcohol (Merck) was used to remove the excess resin on the printed object.

### 2.2. Lignin Extraction

Lignin was extracted from oil palm EFB fibers via an organosolv extraction method, whereby 300 mL of 40–90% FA was added to 10 g of EFB, maintaining an EFB-solution ratio of 1:30. Using a magnetic stirrer, the solution was stirred at 95 °C for 2 h. Next, the solution was filtered using a vacuum filter to remove the pulp from the organosolv lignin. Subsequently, the isolation was carried out using rotary evaporator (RE 600 with VR 300 vacuum controller, Yamato Scientific Co. Ltd., Tokyo, Japan). The resulting black liquor was evaporated to separate the organosolv solution from the extracted lignin. Briefly, a round-bottomed flask was half-filled with lignin organosolv and then placed in a 96 °C water bath. The flask was connected to the evaporator using a clip, and the evaporator was left to operate until the solvent was completely removed. The isolated lignin was then repeatedly washed with water and centrifuged multiple times to remove the excess formic acid. Subsequently, the lignin was recovered via oven-drying.

### 2.3. Preparation of the Photo-Curable Resin Composites

In anticipation of the filler-reinforcement in photo-curable PU, lignin-graphene was prepared with the 10 wt% of graphene nanoplatelets (G) mixed continuously with the extracted organosolv lignin using a homogenizer for 30 min (IKA T 25 Ultra-Turrax Digital High-Speed Homogenizer). Afterwards, photo-curable PU resin was mixed with lignin and lignin-graphene at a different weight ratio (0.2, 0.4, 0.6, 0.8, 1.0, and 3.0 wt%). As a reference, PU was homogenized with 0.02 wt% of graphene, equivalent to the graphene comprised in PU-0.2Lignin/G. The resin compounds were homogeneously mixed using a homogenizer for 10 min or until the particles were well dispersed. The prepared resin compounds were kept in the dark container until further use.

### 2.4. Stereolithography 3D Printing

The UV curable polyurethane with the lignin-graphene mixture was then added into a Duplicator 7 V1.5 by Wanhao 3D printer—a digital light-processing (DLP) system with a 405 nm UV lamp as the curing agent. The STL file model was followed by standard tensile specimen according to the ASTM D638 type IV with the slight modification on the thickness of the tensile specimen, which was set at 0.5 mm, width 7.5 mm, and neck length 24 mm. The density was measured by the dimension and weight of printed samples. Prior to curing under UV light of wavelength 405 nm, the final product was washed in an isopropyl alcohol solution to remove the excess polyurethane resin.

### 2.5. Characterization

The extracted acid-soluble and insoluble lignin was measured by the compositional analysis of TAPPI T222 os-06 (2006) standard. The morphological structure of the sample before and after processes was observed by a variable pressure scanning electron microscope, VPSEM (Merlin Compact, Zeiss Pvt Ltd., Oberkochen, Germany). To determine the types of functional groups present in the lignin as well as the effectiveness of the recovery process, the oil palm EFB fibers and lignin were characterized via Fourier transform infrared spectroscopy (FTIR, Bruker, Billerica, MA, USA) at a resolution of 1 cm^−1^ in the range of 4000 to 650 cm^−1^. Meanwhile, Fourier transform nuclear magnetic resonance (FT-NMR) was performed to analyze the molecular structure of the lignin. In this process, the lignin was dissolved in dimethyl sulphoxide-d6 (DMSO). The components of the extracted lignin were identified by gas chromatography/mass spectrometry (GC-MS, Agilent 7890 GC/5975 MSD), with ethyl acetate as the solvent and DB-5 the column. The viscosity of the photo-curable resin composites was examine using Brookefield Ametek D1 viscometer with spindle type DV1HA at 100 rpm. The tensile test analysis was performed in the Instron^®^ Electromechanical Universal Testing Systems 3300 Series at 500 mm/min with a load cell of 1 kN, following which the tensile strength and elongation were recorded. Nanoindentation behavior of the materials was performed by Nano TestTM (Micro Materials, Wrexham, UK) at the maximum load of 50 mN. Tensile test and nanoindentation analyses were repeated five times for each parameter.

## 3. Results and Discussion

### 3.1. Optimization of Organosolv Lignin Extraction

**Figure 1:**
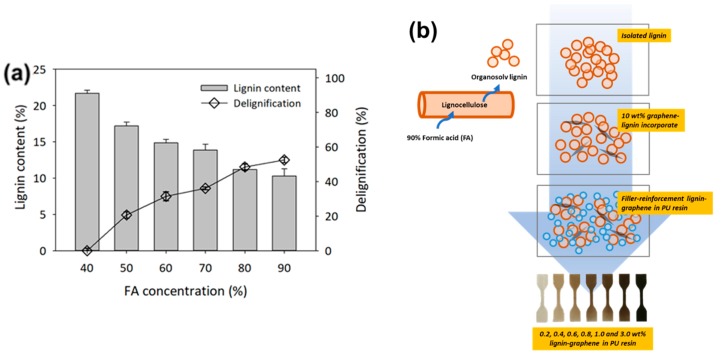
(**a**) The effect of different formic acid (FA) concentration on delignification of empty fruit bunch (EFB) (EFB to aqueous ratio: 1:30, Operating temperature: 90 °C, FA concentration: 40–90%) and (**b**) the further techniques involved in the utilization of organosolv lignin-graphene nanoplatelets with photo-curable polyurethane (PU).

In the preliminary study, the control parameters of FA concentrations on lignocellulosic fractionation were conducted early to maximize lignin that can be extracted. The initial lignin content for the untreated oil palm EFB fibers was recorded at 21.7 wt%. As seen in Figure 1, the amount of acid-soluble lignin extracted from oil palm EFB fibers was increased with the increment of FA concentration. The lignin isolation shows the highest activity at 90% of FA, which extracted ~52.6% from the total lignin in oil palm EFB fibers. Although delignification is the method of breaking down the chemical structure of lignin to make it soluble in a liquid, formic acid has a higher rate of results of dissolution when compared to acetic acid or others with longer carbon chains attributable to the decrease in lignin solubility with increasing alkyl chain length [17,18]. Thus, at higher FA concentration, it provided an effective lignin dissolution through cleavage of ether bonds in lignin macromolecules using acid [19]. The extracted lignin was further isolated by rotary evaporator, repeatedly washed with water to remove the excess formic acid and further modification with graphene nanoplatelets as filler-reinforcement in photo-curable polyurethane resin in the next stages, as seen in Figure 1a.

**Figure 2:**
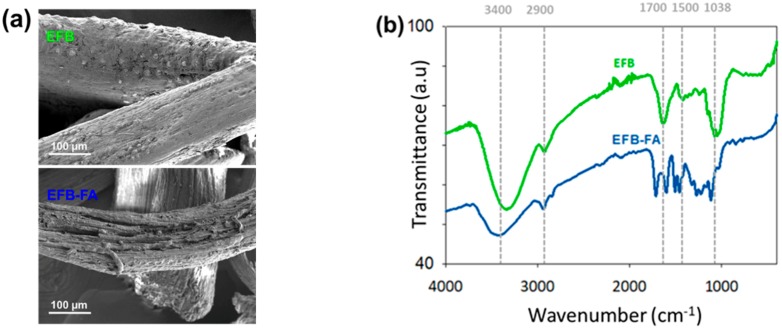
The (**a**) micrograph of VPSEM and (**b**) FTIR spectrums of oil palm EFB fibers before and after the organosolv lignin extraction.

The oil palm EFB fibers show significantly morphological changes after the organosolv lignin extraction, as seen in Figure 2a. The untreated oil palm EFB fibers indicate silica bodies covering the surface structure of the fibers. The treated fibers after organosolv lignin extraction exposed the rough surface structure, an exposed lumen, and noticeable micro cavities due to the removal of lignin and dislodgement of the silica bodies [20]. Additionally, as the advantage of the organosolv extraction, the separation of FA-lignin through rotary evaporator provided 89.9% of the acid recovery, which can be used for the next cycle of the organosolv extraction.

As seen in Figure 2b, the FTIR spectrum at wavenumber of 3400 cm^−1^ exhibited the greater abundance of O–H bonds in oil palm EFB fibers relative to the glucose monomers of cellulose which was significantly changed in organosolv pulp of oil palm EFB fibers and extracted organosolv lignin [20]. Additionally, the peak at 1371 cm^−1^ was present in oil palm EFB fibers but not in lignin is because of alterations in the C–H bond following the removal of cellulose and hemicellulose. Meanwhile, the intensity at 2900 cm^−1^ denoted the C–H of the methyl (CH_2_) and methylene (CH_3_) groups in the monomer of each compound [21]. This finding was also consistent with the fact that the molecules that made up lignocellulose were aromatic and, accordingly, contained a small amount of alkane groups. Typically, the double bond of aromatic carbonyl (C=O) group, C=C aromatic group and ether linkages were observed at 1700 cm^−1^, 1500–1600 cm^−1^, and 1038 cm^−1^ were abundant in lignin, cellulose, and hemicellulose [22,23]. In comparison, syringyl and guaiacyl units of lignin can be identified at 1117 cm^−1^ and 1271 cm^−1^ [24,25].

### 3.2. Characterization of Organosolv Lignin

**Figure 3:**
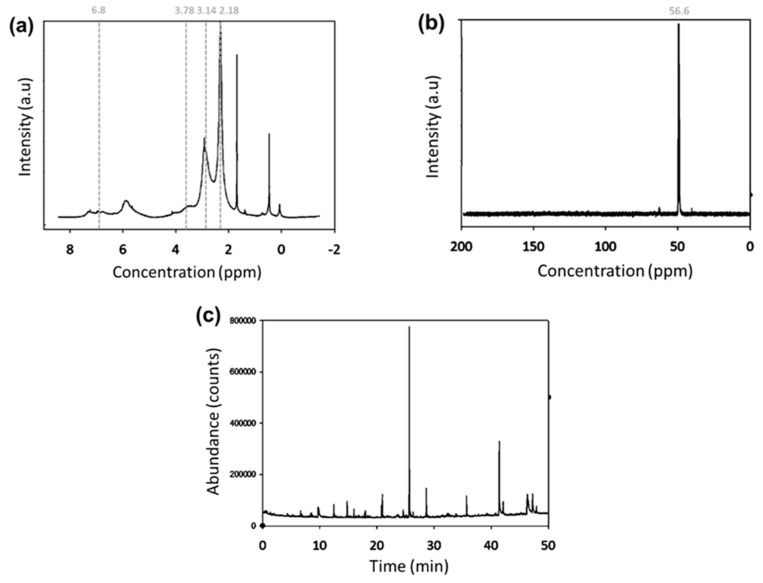
Chemical characterization of organosolv lignin extracted through (**a**) ^1^H NMR, (**b**) ^1^C NMR, and (**c**) GC-MS spectrums.

The ^1^H NMR spectrum of extracted organosolv lignin is shown in Figure 3a. The 3.14 ppm peak shows the presence of hydrogen C-β in β-1 and β-β was stronger than that of the ether linkage because the ether linkages were the easiest to hydrolyze during the formic acid organosolv process [26]. Peaks at 3.758–3.763 ppm revealed the presence of methoxyl proton (–OCH_3_). The peak at 8.133 ppm revealed the presence of aromatic protons in guaiacyl (G) and syringyl (S) units and 6.524–6.826 ppm aromatic protons in the syringyl-propane units verify the presence of the main monomers of lignin in the sample. Furthermore, the stronger signal at 6.8 ppm relative to that at 7.0 ppm indicates that the lignin contained more syringyl than guaiacyl monomers [27,28]. Other peaks were detected at (1) 7.792–7.868 ppm ortho-hydrogen within the carbonyl groups; (2) peak at 4.898 ppm β-H within the β-O-4 linkages; (3) 2.18–2.216 ppm phenolic protons in lignin; (4) 1.272 ppm and 0.861–0.895 ppm methylene and methyl groups respectively within the lignin side chains; as well as (5) 2.500–2.216 ppm protons in the solvent (DMSO-d6) [22,26,29,30].

The ^1^C NMR provided in Figure 3b shows significant signals at 56.56 ppm, indicating the presence of –OCH_3_ in the syringyl units, 39.72–40.97 DMSO-d6 (i.e., the solvent), as well as 29.34 β-methylene in the n-propyl side chains. On the contrary, the absence of peaks at 57–103 ppm implied that the lignin did not contain a noticeable amount of polysaccharide. It may be concluded that the ester and ether bonds in lignin and hemicellulose have been successfully cleaved during organosolv extraction [28].

As seen in Figure 3c, the GC-MS spectrum revealed the presence of a phenolic molecule and 1,1-bifenil-2,3-diol, both of which gave rise to a phenylpropane unit, the building block of lignin [31]. This was attributable to the chemical modification of the β-O-4 linkage during extraction [32]. The abundant presence of these two components also showed that this sample was rich in phenolic molecules. Apart from phenolic monomers, acyclic hydrocarbons like nonadecane, 1-dodecene, 6-tetradecene, hexadecene, (Z), 2-bromononane, tetratetracontane, 2,6-dimethyldecane, and methylnonadecane were also present. Other acyclic components included oxygenated components, such as 1-heptacosanol, oxalic acid, n-hexadecanoic acid, 9,12-octadecadienoic acid (Z,Z)-, octadecanoic acid, and danbromoacetic acid, which were also present in the lignin [33]. The reason behind the presence of n-hexadecanoic acid or palmitic acid in the lignin sample was that the acid is a typical component of the oil within palm fruits [34].

### 3.3. Tensile Strength of 3D-Printed Composites

**Figure 4:**
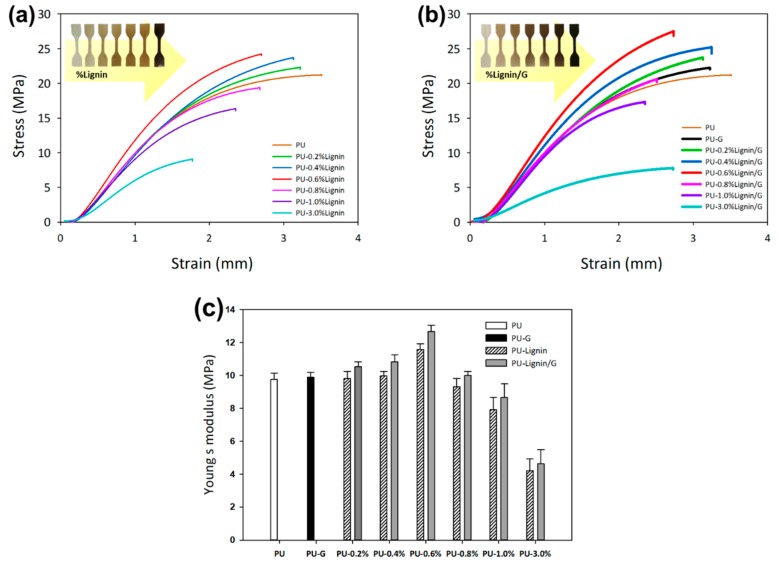
Mechanical properties of the photo-curable PU at stress-strain curves of (**a**) composited with lignin, (**b**) lignin/graphene, and (**c**) Young’s modulus behaviours of the materials.

As seen in the graph in Figure 4a, the composites which contained 1% and 3% of lignin had lower tensile strength than the resin. Meanwhile, the composite whose lignin concentration was 0.6% had a tensile strength close to that of resin. When lignin was added to the PU resin, the proportion of resin decreased; however, the presence of hydroxyl- and phenolic-rich lignin gave rise to a 3-dimensional structure with polyurethane and retained the tensile strength of the [35]. Likewise, the tensile strength of the PU-0.6% Lignin/G composite was much greater than that of the PU resin and lignin incorporated, showing that the mechanical property of the composite was enhanced when the concentration of lignin-graphene increased until 0.8% addition. The increase in lignin-graphene content increased the hardness of the composite and reduced the flexibility of the polymer. The 3D printed composited shown in Figure 4 displays the darkening of the samples with an increment of the lignin and lignin/G. The maximum load the filler-reinforcement was able to attain was 3%; higher loading interrupted the curing behavior of the photo-curable PU resin and defected the orientation of the printed samples.

Figure 4c shows the Young’s modulus of the sample with the increment of lignin and lignin-graphene as well as the data summarized in Table 1. The low loading lignin-graphene shows exceptional performance: as tensile strength increased, the ductility of the materials was compromised [16]. In contrast, the addition of lignin-graphene improved the Young’s modulus of the composite. The high filler-reinforcement load often resulted in particle agglomeration; however, the viscosity measurement tabulated in Table 1 shows slight changes (~0.55% viscosity increase) even at the highest concentration of the additional filler-reinforcement of lignin-graphene [15].

### 3.4. Nanoindentation Behavior of 3D-Printed Composites

**Figure 5:**
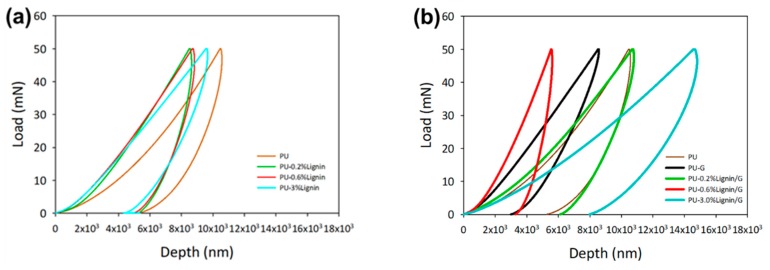
Nanoindentation of the photo-curable PU composited with (**a**) lignin and (**b**) lignin/graphene.

Nanoindentation technique on the photo-curable PU composites has been carried out at the maximum load of 50 mN. The loading-unloading curves of the sample can be seen in Figure 5 and the calculated data is summarized in Table 2. The contribution of lignin as a filler in PU resin provided a minimum reformation of the sample hardness.

In the additional of lignin-graphene, typical loading-unloading behavior in comparison with stress-strain curve was observed, which demonstrated the depth indentation decreased and the curves shifted to the left due to the increment of the hardness [36]. The filler-reinforcement of lignin-graphene showed the highest improvement at 0.6% Lignin/G as the sample provided tremendous enhancement of the hardness at 92.49 MPa (238% increment from unmodified photo-curable PU resin). The integration of graphene nanoplatelets was shown to be the main element for the improvement of the hardness of the PU. The resistance of the graphene towards deformation of the material provided better elasticity [36]. In contrast, the chemical interaction between the isocyanate group and oxygenated groups of PU-graphene provided the compatibility of the lignin-graphene in photo-curable PU resin [14]. However, the increment of the graphene up to 3% showed a similar trend as a stress-strain curve, which exhibited the low hardness profile of the materials.

### 3.5. The Compatibility of PU-Lignin with Graphene

**Figure 6:**
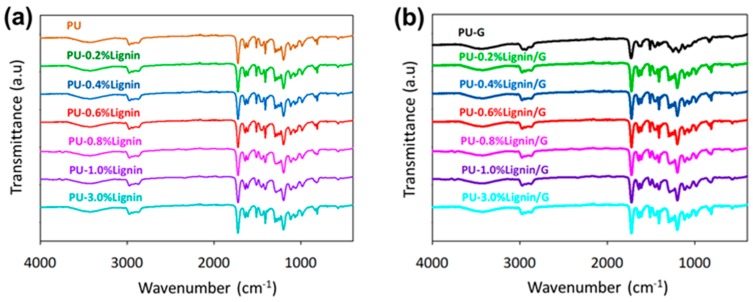
FTIR spectrums of the photo-curable PU composited with (**a**) lignin and (**b**) lignin/graphene.

As seen in Figure 6, the chemical compatibility of photo-curable PU with lignin and graphene was analyzed through FTIR spectrums. In cured PUs, the infrared region indicates fewer isocyanates, discovered at 2312 cm^−1^, suggesting that there is no surplus moisture and isocyanates in pre-polymer reactants [37]. Thus, curing of the polymer is optimal as no major peak was observed at 2312 cm^−1^. The broad peak was observed at 3200–3600 cm^−1^, indicating the presence of alcoholic and phenolic –OH absorptions. Peaks observed at 1400–1600 cm^−1^ indicated the presence of aromatic structures present in the PU [38]. After the addition of the lignin-graphene, a strong interfacial interaction between functional groups of lignin-graphene and polyurethane occurred resulting in better compatibility, suggesting a strong hydrogen bond between polyurethane and lignin-graphene [39].

**Figure 7:**
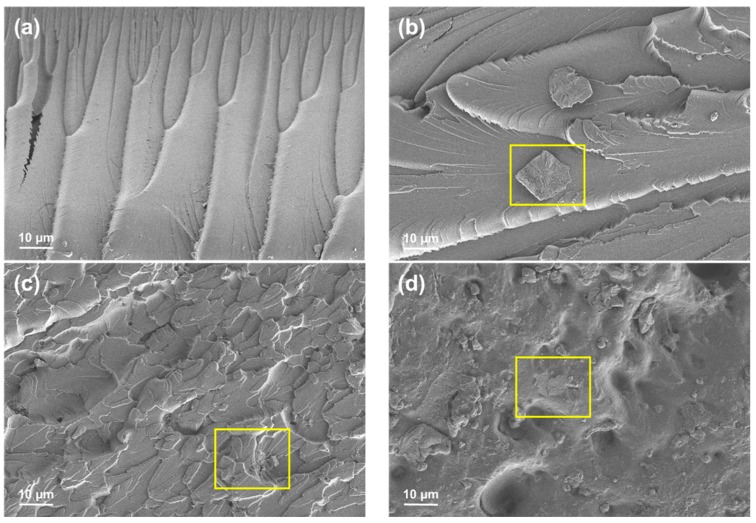
Micrograph images of the fracture surface after tensile testing (**a**) PU, (**b**) PU-G, (**c**) PU-0.6% Lignin/G, and (**d**) top surface of PU-0.6% Lignin/G.

The micrograph of the cross-section 3D-printed composites after tensile testing shows the effect of lignin-graphene in favor of the better compatibility of filler-reinforcement in photo-curable PU, as seen in Figure 7. PU samples indicated a wide line of micro cracks and homogeneously flat surface area. In Figure 7b, graphene nanoplatelets were distinctly spotted on the PU-G surface with non-uniform blending between photo-curable PU. In the addition of lignin-graphene, the blending between PU-lignin-graphene was well distributed in the polymeric matrix [40].

A small poor dispersion of graphene nanoplatelets will provide more concentrated stress locally, affecting the mechanical properties of the material [41]. The clear morphological structure from the top of the PU-0.6% Lignin/G which has been exposed to the UV light, as seen in Figure 7d, exhibited the homogeneity of graphene nanoplatelets with the assistance of lignin as filler reinforcement in photo-curable PU. The synergistic interaction seen in lignin-graphene, lignin-polyurethane, graphene-polyurethane, and lignin-graphene in polyurethane resin plays a major role in the formation of compatible photo-curable resin composites. In comparison with lignin-polyurethane blends, the molecular structure of lignin tends to form a lignin-lignin interaction; the viscosity of the resin composite gradually increases as the composition of the lignin increases, as seen in Figure 1a. Unlike graphene oxide, which has a nanolayer formation, the graphene nanoplatelets particles require proper mechanical homogenization method to distribute in the polyurethane system [36,39,42]. Accordingly, the composition of lignin-graphene in polyurethane system facilitated greater distribution, significantly increasing the mechanical properties of the 3D-printed composites.

## 4. Conclusions

In this study, the extracted lignin at the best condition of organosolv extraction reveals the presence of syringyl, guaiacyl, and hydroxyl molecules, which is highly compatible with the photo-curable PU. The ability of the organosolv lignin as a compatibilizer for graphene nanoplatelets was successfully demonstrated in the mechanical properties of the PU-Lignin/G. Substantial improvement by filler-reinforcement of lignin-graphene in photo-curable PU, identified at 0.6% Lignin/G as the stress-strain and loading-unloading behavior, shows higher tensile strength and the resistance against the deformation of the material.

## Figures and Tables

**Table 1:** The effect of resin viscosity towards the tensile Stress-Strain and Young’s modulus of photo-curable PU composited with lignin and lignin/graphene.

Sample	Viscosity (cP)	Stress (MPa)	Strain (mm)	Young’s Modulus (MPa)
PU	50.63 ± 0.05	21.15 ± 0.21	3.51 ± 0.02	9.77 ± 0.15
PU-0.2%Lignin	50.72 ± 0.07	22.19 ± 0.11	3.21 ± 0.05	9.81 ± 0.13
PU-0.6%Lignin	50.76 ± 0.09	24.50 ± 0.32	2.69 ± 0.03	11.59 ± 0.14
PU-3.0%Lignin	50.91 ± 0.04	9.19 ± 0.22	1.77 ± 0.06	4.21 ± 0.09
PU-G	50.64 ± 0.03	22.56 ± 0.29	3.22 ± 0.09	9.89 ± 0.13
PU-0.2%Lignin/G	50.73 ± 0.04	23.87 ± 0.24	3.11 ± 0.02	10.53 ± 0.16
PU-0.6%Lignin/G	50.76 ± 0.04	27.35 ± 0.30	2.73 ± 0.05	12.68 ± 0.17
PU-3.0%Lignin/G	50.93 ± 0.06	8.39 ± 0.16	7.80 ± 0.03	4.63 ± 0.13

**Table 2:** The loading-unloading behavior of photo-curable PU composited with lignin and lignin/graphene.

Sample	Max Depth (nm)	Plastic Depth (nm)	Hardness (MPa)
PU	10,573 ± 15	8708 ± 12	27.33 ± 0.3
PU-0.2%Lignin	9665 ± 3	8181 ± 7	38.21 ± 0.3
PU-0.6%Lignin	8830 ± 11	5079 ± 8	38.96 ± 0.2
PU-3.0%Lignin	9655 ± 7	8011 ± 6	38.12 ± 0.1
PU-G	8573 ± 5	6154 ± 4	54.51 ± 0.4
PU-0.2%Lignin/G	10,799 ± 8	8715 ± 5	27.29 ± 0.3
PU-0.6%Lignin/G	5609 ± 12	4714 ± 8	92.49 ± 0.4
PU-3.0%Lignin/G	14,801 ± 8	11,951 ± 11	14.54 ± 0.3
